# Acetaldehyde-Derived Advanced Glycation End-Products Promote Alcoholic Liver Disease

**DOI:** 10.1371/journal.pone.0070034

**Published:** 2013-07-26

**Authors:** Nobuhiko Hayashi, Joseph George, Masayoshi Takeuchi, Atsushi Fukumura, Nobuyuki Toshikuni, Tomiyasu Arisawa, Mikihiro Tsutsumi

**Affiliations:** 1 Department of Hepatology, Kanazawa Medical University, Uchinada, Ishikawa, Japan; 2 Department of Advanced Medicine, Kanazawa Medical University, Uchinada, Ishikawa, Japan; 3 Department of Gastroenterology, Kanazawa Medical University, Uchinada, Ishikawa, Japan; University of California, Merced, United States of America

## Abstract

**Background:**

Chronic ingestion of ethanol increases acetaldehyde and leads to the production of acetaldehyde-derived advanced glycation end-products (AA-AGE). We evaluated the toxicity of AA-AGE on hepatocytes and studied the role of AA-AGE in the pathogenesis of alcoholic liver disease (ALD).

**Methods:**

Rat hepatocyte cultures were treated with N-ethyllysine (NEL) or AA-AGE and the cell viability was evaluated using MTT assay. Male Wistar rats were fed with liquid diet containing 5% ethanol for 8 weeks following normal diet for another 12 weeks. A group of animals was sacrificed at 4th, 6th, and 8th week and the remaining animals at 12th, 14th, 16th, 18th, and 20th week. The liver sections were stained for AA-AGE and 4-hydroxy-2-nonenal (4-HNE). Liver biopsy obtained from ALD patients was also stained for AA-AGE and 4-HNE.

**Results:**

Hepatocyte viability was significantly reduced in cultures treated with AA-AGE compared to NEL treated or control cultures. Severe fatty degeneration was observed during chronic administration of ethanol increasing from 4–8 weeks. The staining of AA-AGE and 4-HNE was correlated with the degree of ALD in both rat and human. In rats, hepatic fatty degeneration was completely disappeared and the staining for both AA-AGE and 4-HNE returned to normal at 12th week of abstinence. Staining for AA-AGE and 4-HNE was completely absent in normal human liver.

**Conclusions:**

The data demonstrated that AA-AGE is toxic to hepatocytes, but not NEL. Chronic ethanol ingestion produces AA-AGE and reactive oxygen species that contribute to the pathogenesis of ALD. Abstinence of alcohol results in complete disappearance of both AA-AGE and 4-HNE along with fatty degeneration suggesting that AA-AGE plays a significant role in the pathogenesis of ALD.

## Introduction

The pathogenesis of alcoholic liver disease (ALD) is a dynamic process triggered by complex interactions between metabolic intermediates of alcohol, inflammation and immune responses from cellular injury [1], [2]. Since hepatocytes are the primary site of alcohol detoxification, its major toxic metabolic intermediate, acetaldehyde causes direct hepatocyte damage and also forms adducts with proteins and DNA [3], [4]. Acetaldehyde produces two distinct groups of adducts depends on the prevailing conditions. The first group is formed under reducing conditions and comprises N-ethyl amino groups. The second group is formed under non-reducing conditions and consists of a wide spectrum of adducts that are not well characterized. The initial step in the formation of the second group of adducts is often to form a Schiff base, which then undergoes a series of rearrangements and further reactions to generate different kinds of adducts [5]. N-ethyllysine (NEL) is a reduced form of protein-acetaldehyde adduct, which has been detected in the livers of patients with alcoholic liver disease and in experimental animals fed with alcohol [6], [7] suggesting that NEL may play a role in the pathogenesis of ALD.

The biochemical and pathological role of non-enzymatic glycation of proteins by reduced sugars such as glucose has become increasingly evident in the pathogenesis of various diseases [8], [9]. It is now well established that early glycation products undergo progressive modification *in vivo* to form irreversible cross-links over time, after which the molecules are known as advanced glycation end-products (AGEs) [10]. AGEs have been implicated in the development of many of the pathological sequelae of diabetes and aging, such as atherosclerosis, stroke, and renal insufficiency [8]−[11]. AGEs also play a significant role in neuro-degenerative disorders such as Alzheimer’s disease and Parkinson's diseases as well as in heart diseases, cancer, and non-alcoholic steatohepatitis [12]−[−16].

Based on our previous studies [17]−[19] we proposed a pathway for the formation of acetaldehyde-derived advanced glycation end-products (AA-AGE) by the Maillard reaction *in vivo* [20]. It was also demonstrated that AA-AGE is produced purely from acetaldehyde and has properties similar to adduct formed by non-enzymatic glycation by sugar (AGE adducts) [5]. We hypothesize that NEL pathway for reaction of Amadori compounds could serve as a physiologically relevant mechanism for averting potentially serious consequences of the AA-AGE formation. The present study was aimed to examine the association of AA-AGE and pathogenesis of alcoholic liver disease. The toxicity of AA-AGE and NEL was evaluated using cultured rat hepatocytes. The intralobular distribution of AA-AGE was monitored during chronic ethanol administration and also during alcohol abstinence and co-evaluated with the progression of ALD and production of reactive oxygen species (ROS).

## Materials and Methods

### 1. Preparation of AA-AGE-BSA and NEL-BSA

AA-AGE was prepared as described previously [20]. Briefly, 25 mg/ml of bovine serum albumin (BSA) was incubated with 0.1 M acetaldehyde and 5 mM diethylene triaminepenta-acetic acid in 0.2 M phosphate buffer (pH 7.4) at 37°C for 7 days under sterile conditions. As control, BSA was incubated under the same conditions without acetaldehyde. The control BSA and AA-AGE were purified using a PD-10 desalting column chromatography and dialysis against phosphate buffered saline (PBS). The preparation of NEL-BSA was also described before [20]. In brief, 50 mg/ml of BSA was incubated with 50 mM acetaldehyde and 150 mM sodium cyanoborohydride in 2 ml of 0.2 M phosphate buffer (pH 7.4) at 37°C for 24 h. The product was purified using PD-10 column chromatography and dialysis against PBS. The preparation was then passed through Zeta-Pore filter (Cuno Co., Tokyo, Japan) to remove endotoxin. Protein concentration of the preparations was determined with Bio-Rad DC protein assay using BSA as standard.

### 2. Preparation and Purification of Anti-AA-AGE and NEL Antibodies

Anti-AA-AGE antibody was prepared and purified as described previously [20]. Briefly, 25 ml of rabbit AA-AGE antiserum was applied to a Sepharose 4B column (GE Healthcare, Piscataway, NJ) coupled with AA-AGE-BSA. After extensive washing with PBS, the adsorbed fractions were eluted with 20 mM sodium phosphate buffer containing 1 M potassium thiocyanate (pH 7.4). The anti-AA-AGE antibody fractions were pooled, concentrated using Centriprep-10 (Millipore), and then passed through a PD-10 column. The antibody thus obtained was loaded on to a Sepharose 4B column (GE Healthcare) coupled with NEL-BSA and then washed with PBS to obtain the unadsorbed fraction (anti-AA-AGE antibody). The unadsorbed fractions were pooled, concentrated with Centriprep-10, and then passed through a PD-10 column. The purified AA-AGE antibody was employed in this study.

The anti-NEL antibody was also prepared and purified as described before [20]. In brief, 25 ml of rabbit NEL antiserum was applied to a Sepharose 4B column coupled with NEL-BSA. After extensive washing with PBS, the adsorbed fractions were eluted with 20 mM sodium phosphate buffer containing 1 M potassium thiocyanate (pH 7.4). The anti-NEL antibody fractions were pooled, concentrated using Centriprep-10, and passed through a PD-10 column and used in the current study.

### 3. Isolation and Culture of Hepatocytes from Rat Liver

Hepatocytes were isolated from 8 weeks old male Wistar rats weighing around 250 g employing collagenase digestion method [21]. In brief, the animals were anesthetized with intraperitoneal injection of 0.4 ml sodium pentobarbital (50 mg/ml), sterilized the abdomen with 70% ethanol, dissected the skin and peritoneum, cannulated the portal vein with 20 gauge angiocath and tied. Then the inferior vena cava was cannulated through the heart. The liver was perfused with Ca^++^ and Mg^++^ free Hanks balanced salt solution at a flow rate of 10 ml/min for 10 min followed by 0.05% collagenase in serum free Dulbecco’s Modified Eagles Medium (DMEM, Nissui Co. Tokyo, Japan) for 20 min. The perfused livers were excised, removed Glisson’s capsule, cut into small pieces and dissociated in 10–15 ml serum free DMEM. The cell suspension was filtered through double layer sterile cotton gauze and centrifuged at 1200 rpm for 5 min. Any inclusion of non-parenchymal cells in the preparation was removed by Percoll density gradient centrifugation. The separated hepatocytes were collected and suspended in 10 ml of serum free DMEM and washed twice through centrifugation. Trypan blue exclusion test revealed 90–95% viability of purified hepatocytes. The isolated hepatocytes were counted using a hemocytometer and suspended in DMEM supplemented with 10% fetal bovine serum (FBS) (Invitrogen, Carlsbad, CA) and antibiotics (penicillin 100 units/ml and streptomycin 100 µg/ml).

The hepatocytes were then seeded at a density of about 10^4^ cells (in 200 µl) into each well of a 96-well culture dish and placed in a humidified incubator containing 5% CO_2_ on air at 37°C. After 6 h the culture medium was replaced with fresh medium.

### 4. Effect of AA-AGE and AA-AGE Antibody on Hepatocyte Viability

The 3-(4,5-dimethylthiazolyl–2)-2,5-diphenyltetrazolium bromide (MTT) assay [22] was performed to determine the viability of hepatocytes after treatment with AA-AGE or NEL and after neutralization of AA-AGE using anti-AA-AGE antibody [23]. At 24 h after seeding, the culture media was removed and the hepatocytes were washed twice with serum-free DMEM in order to remove any non-adherent or dead cells. Then the cells were treated with DMEM containing 25 µg/ml of AA-AGE or NEL or unmodified BSA or 25 µg/ml of AA-AGE and 25 µg/ml of AA-AGE antibody (final concentration) supplemented with 2% FBS and incubated for 48 h in a CO_2_ incubator. Six sets in duplicate were prepared in each group. The MTT assay was performed as per manufacturer’s instructions (Chemicon International, Temecula, CA). The MTT formazan was dissolved by the addition of isopropanol and the final blue color was measured using an ELISA reader (Bio-Rad, Hercules, CA). The percentage decrease of cell viability in AA-AGE or NEL treated samples were calculated based on 100% viability of cells treated with unmodified BSA.

### 5. Chronic Administration of Ethanol in Rats

All animal experiments were carried out with the *Guide for the Care and Use of Laboratory Animals* published by the US National Institutes of Health (NIH Publication No. 86–23, revised 1996). The protocol was also approved by the Animal Care and Research Committee of Kanazawa Medical University on the Ethics of Animal Experiments. About 5 weeks old 30 male Wistar rats (body weight 160±15 g) were divided into two groups of 15 rats each. One group was received 5% ethanol containing liquid diet (36% of total calories) and the second group was pair-fed with control diet in which ethanol was replaced isocalorically with carbohydrate [24]. The animals were sacrificed under anesthesia at 4th, 6th, and 8th week along with control animals and the blood was collected. The livers were quickly removed and the median lobe was cut into 3 mm pieces and fixed in 10% phosphate-buffered formalin for histopathology and the remaining liver tissue was flash frozen in liquid nitrogen. The formalin fixed liver tissues were processed in an automatic tissue processor optimized for liver, embedded in paraffin blocks, and cut into sections of 5 µm thickness. The sections were stained with hematoxylin and eosin (H&E) as per standard protocol. The stained sections were examined under an Olympus BX50 microscope attached with DP 71 digital camera (Olympus Corporation, Tokyo, Japan) and photographed.

### 6. Liver Biopsy from Patients with Alcoholic Liver Disease

All patients involved in the study were admitted to the Kanazawa Medical University hospital for diagnosis and treatment. The procedure was reviewed and approved by the Ethical and Clinical Investigation Committee of the Kanazawa Medical University. Written consent was obtained from each patient prior to the procedure after full explanation of the purpose of the study. The patients had ingested more than 80 g alcohol every day over 10 years. The serum markers for hepatitis B virus (HBV) and hepatitis C virus (HCV) were negative in all selected patients. Liver biopsy was performed on 25 patients. Control liver specimens were obtained from healthy donors during surgical procedures. The liver biopsy sections were stained for H&E and evaluated for ALD and steatosis.

### 7. Measurement of ALT and AST in Serum

The blood was allowed to clot for 3−5 hrs and serum was separated following conventional method. Serum alanine transaminase (ALT) and aspartate transaminase (AST) were measured using an auto-analyzer.

### 8. Measurement of Glutathione (GSH+GSSG) in the Liver

Total glutathione (GSH+GSSG) present in the liver tissue of untreated control rats and during chronic administration of ethanol as well as during alcohol abstinence was determined using glutathione assay kit procured from Sigma (St. Louis, MO, USA). This kit is designed to measure the level of total glutathione (GSH+GSSG) in biological samples. In brief, the liver tissue was flash frozen in liquid nitrogen immediately after sacrifice of the animals and stored at −80°C until assayed. About 100 mg of liver frozen tissue was homogenized in 1 ml of ice cold 50 mM Tris-HCl buffer (pH 8) containing 150 mM NaCl, 1 mM EDTA, and 1% Triton X-100. Then 50 µl of the homogenate was treated with 150 µl 5% 5-sulfosalicylic acid solution and vortexed well. It was allowed to stand on ice for 10 min and homogenized at 12,000 × *g* for 10 min at 4°C. The supernatant was 1∶1 diluted with distilled water and the samples in triplicate were assayed for GSH concentrations in 96 well microplate. About 10 µl of diluted sample was mixed with 150 µl of working mixture containing 100 mM potassium phosphate buffer (pH 7.0), 5,5′−dithiobis-2-nitrobenzoic acid (DTNB), and glutathione reductase. It was incubated for 5 min at room temperature followed with the addition of 50 µl NADPH solution (0.16 mg/ml) using a multichannel pipette and mixed well by pipetting up and down. It was allowed to stand for 5 min at room temperature and the resultant yellow color was read on an ELISA plate reader at 412 nm.

### 9. Immunohistochemical Staining for AA-AGE and 4-HNE

The liver sections obtained in the above study were subjected to immunohistochemical staining for AA-AGE and 4-hydroxy-2-nonenal (4-HNE) to examine production of AA-AGE and ROS during ethanol intake. Furthermore, about 5 weeks old 30 male Wistar rats were administered ethanol-containing liquid diet for 8 weeks and then gave control liquid diet for 12 weeks. Five animals each were sacrificed at 4, 6, 8, 10, and 12th weeks from the beginning of administration of control liquid diet and the blood and livers were collected. A group of age matched control animals were also sacrificed at 12 weeks. The liver tissue sections were stained with H&E and examined for the decrease of ALD and steatosis during abstinence. The animal and human tissue sections were then stained immunohistochemically for AA-AGE and 4-HNE with immunoaffinity-purified antibodies using broad-spectrum histostain kit (Invitrogen, Carlsbad, CA, USA). The AA-AGE antibody was prepared as described before [20] and the 4-HNE antibody (MHN-100P) was procured from Nikken Seil Co., Shizuoka, Japan. The final stain was developed using 3% 3-amino-9-ethylcarbazole (AEC) in N, N-dimethylformamide. The stained sections were examined using an Olympus microscope attached with a digital camera and photographed. The staining intensity in 10 randomly selected microscopic fields were quantified using Image-Pro Discovery software (Media Cybernetics, Silver Spring, MD, USA) and presented as percentage square microns.

### 10. Effect of AA-AGE on Rat Hepatic Stellate Cells

In order to study whether AA-AGE could induce oxidative stress and produce ROS *in vitro*, we cultured rat hepatic stellate cells and treated with AA-AGE for 24 h. Hepatic stellate cells were isolated from 12 month-old albino rats of Wistar strain and cultured as described before [25]. The confluent stellate cells were frozen in liquid nitrogen and passed twice. The frozen cells were then cultured in 50∶50 mixture of DMEM and Ham's F12 medium (Invitrogen, Carlsbad, CA, USA) supplemented with 10% FBS and antibiotics in a humidified incubator containing 5% CO_2_ on air at 37°C. About 80% confluent stellate cells were harvested using TrypLE Express (Invitrogen, Carlsbad, CA, USA) and sub-cultured into 4-well glass microscopic chamber slides (Nalge Nunc Lab-Tek, NY) coated with collagen-fibronectin. After 24 hrs, the media were replaced with a reduced serum medium (2%) containing either 5 µg/ml or 10 µg/ml AA-AGE (final concentration) and incubated for another 24 hrs. The media were removed and the cells were fixed in 1∶1 methanol and ethanol mixture at −20°C for 10 min. The cells were then washed twice with cold PBS and stained for 4-HNE as described above. The intensity of 4-HNE staining was quantified using Image-Pro Discovery software (Media Cybernetics, Silver Spring, MD, USA) and presented as percentage square microns.

### 11. Statistical Analysis

Arithmetic mean and standard deviation (Mean ± S.D.) were calculated for all the data. The data were analyzed either by using one way analysis of variance (ANOVA) or multiple group comparison depended on the situation. Student’s *t*-test was also used on certain places. Control mean values were compared with experimental mean values on different weeks of treatment and abstinence groups using least significant difference method. A value of *P*<0.05 was considered as statistically significant.

## Results

### 1. Treatment with AA-AGE Significantly Reduced Hepatocyte Viability

**Figure 1:**
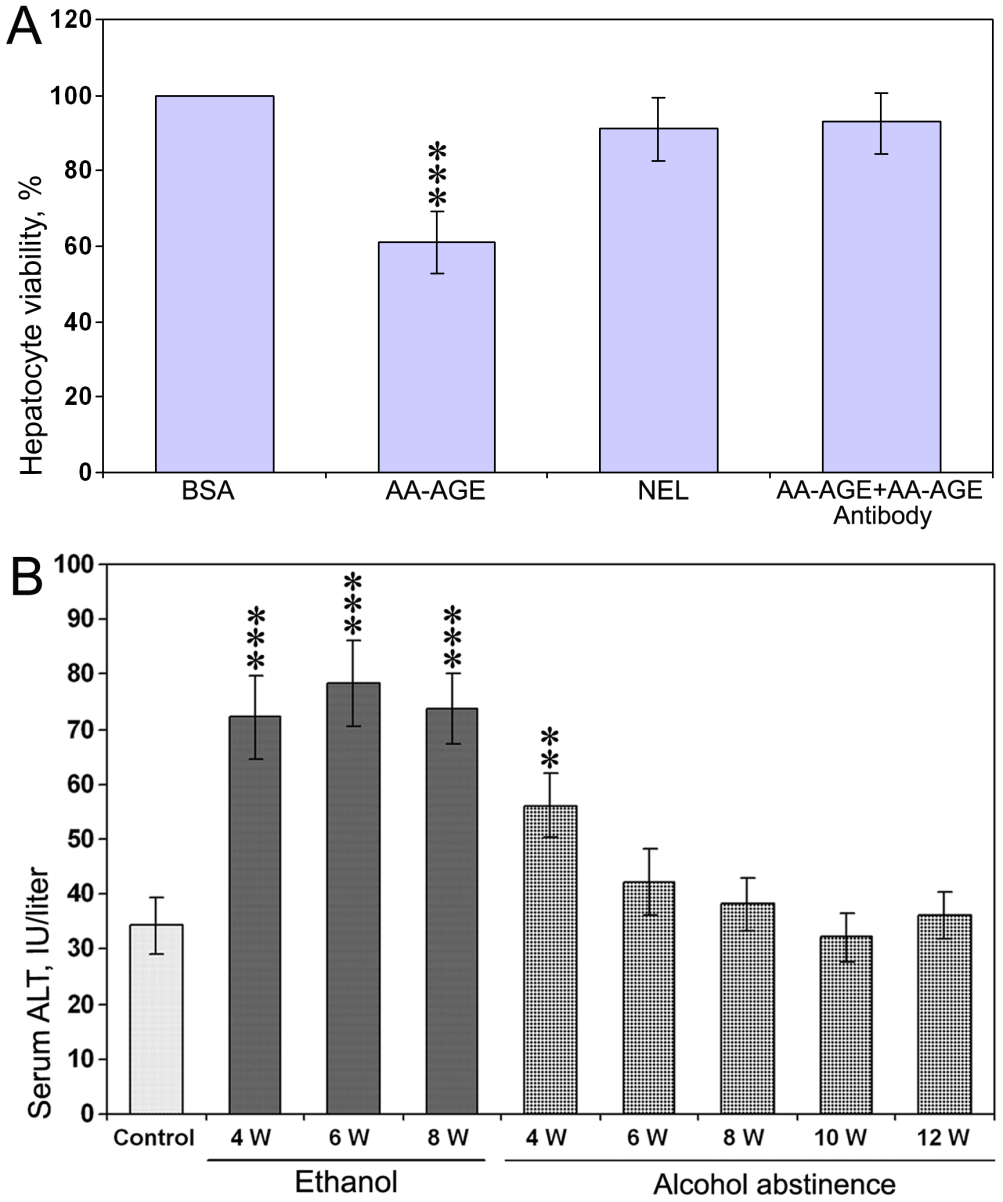
Effect of AA-AGE on viability of cultured rat hepatocytes. (A) The 24 h old hepatocyte cultures were treated with media containing 25 µg/ml of AA-AGE or NEL and incubated for 48 h. Viability of hepatocytes cultured with AA-AGE was significantly decreased compared to the hepatocytes cultured with NEL. Neutralization of AA-AGE with AA-AGE antibody resulted in complete prevention of hepatocyte cell death induced by AA-AGE. ***P<0.001 compared to the NEL treated cultures by Student’s *t*-test. (B) Alanine transaminase levels in rat serum after the start of chronic administration of alcohol for 8 weeks and also after abstinence. **P<0.01 and ***P<0.001 compared to control by ANOVA (n = 5).

The results of MTT assay on viability rate of rat hepatocytes after treatment with AA-AGE and NEL are presented in Figure 1A. There was a significant decrease (*P*<0.001) in hepatocyte viability at 48 h after treatment with 25 µg/ml of AA-AGE compared to unmodified BSA. The rate of cell viability was decreased to 61%. On the other hand, viability of hepatocytes cultured with 25 µg/ml of NEL was 91% compared to unmodified BSA (Fig. 1A) and the decrease was not significant. The decrease of hepatocyte viability in AA-AGE treated samples was also significantly lower (*P*<0.001) compared to NEL treated samples. Neutralization of AA-AGE using anti-AA-AGE specific antibody in the culture media resulted in a complete prevention hepatocyte cell death induced by AA-AGE (Fig. 1A). This experiment also proved that the purified AA-AGE antibody is specific to AA-AGE and has the ability to neutralize AA-AGE.

### 2. Serum ALT and AST Levels after Chronic Administration of Ethanol

Serum ALT levels during the period of chronic administration of ethanol and after abstinence of alcohol are presented in Figure 1B. Serum ALT levels were significantly (*P*<0.001) higher on weeks 4, 6, and 8 after chronic administration of ethanol and also at the 4th week after alcohol abstinence (*P*<0.01), but returned to normal level afterwards. There was no significant alteration in serum AST levels at any time point during course of the study (data not shown).

### 3. Glutathione Levels in Rat Liver Treated with Alcohol

**Figure 2:**
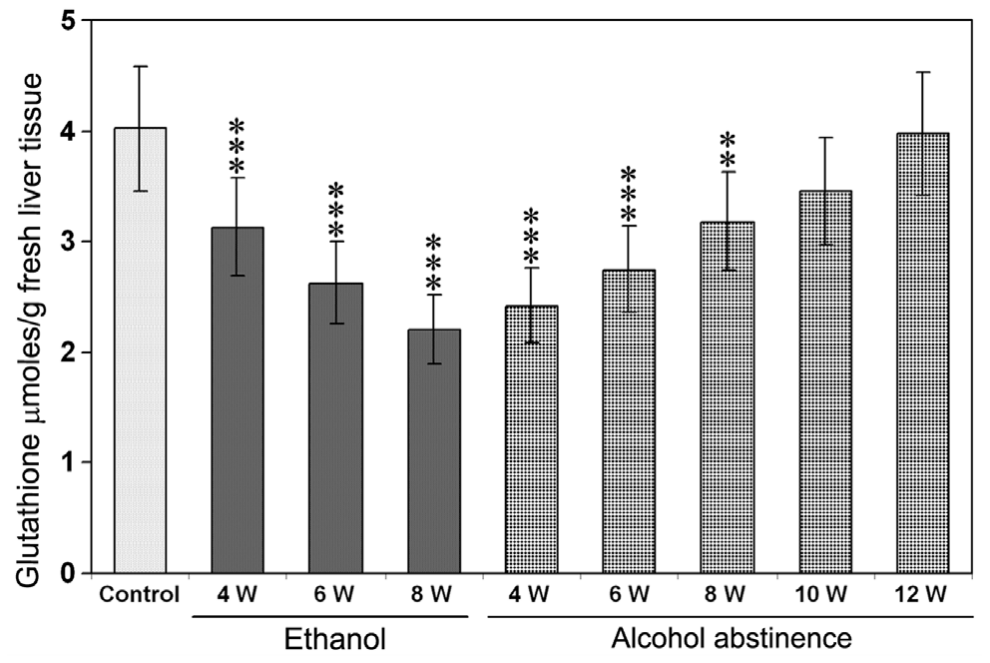
Glutathione levels in the liver during chronic administration of ethanol and after alcohol abstinence. A significant decrease was observed in total glutathione levels (GSH+GSSG) at 4th, 6th, and 8th week of ethanol administration through liquid diet. **P<0.01 and ***P<0.001 compared to the untreated control by ANOVA (n = 5).

The total glutathione levels (GSH+GSSG) measured in the liver samples of control rats, during chronic administration of alcohol, and also during alcohol abstinence are presented in Figure 2. Glutathione levels were significantly decreased (*P*<0.001) at weeks 4, 6, and 8 with a maximum decrease at week 8 after the start of alcohol administration through liquid diet. The decreased glutathione levels were returned to normal at week 10 from the beginning of alcohol abstinence (Fig. 2).

### 4. Histopathological Evaluation of Alcoholic Liver Disease during Chronic Ingestion of Ethanol and in Abstinence

**Figure 3:**
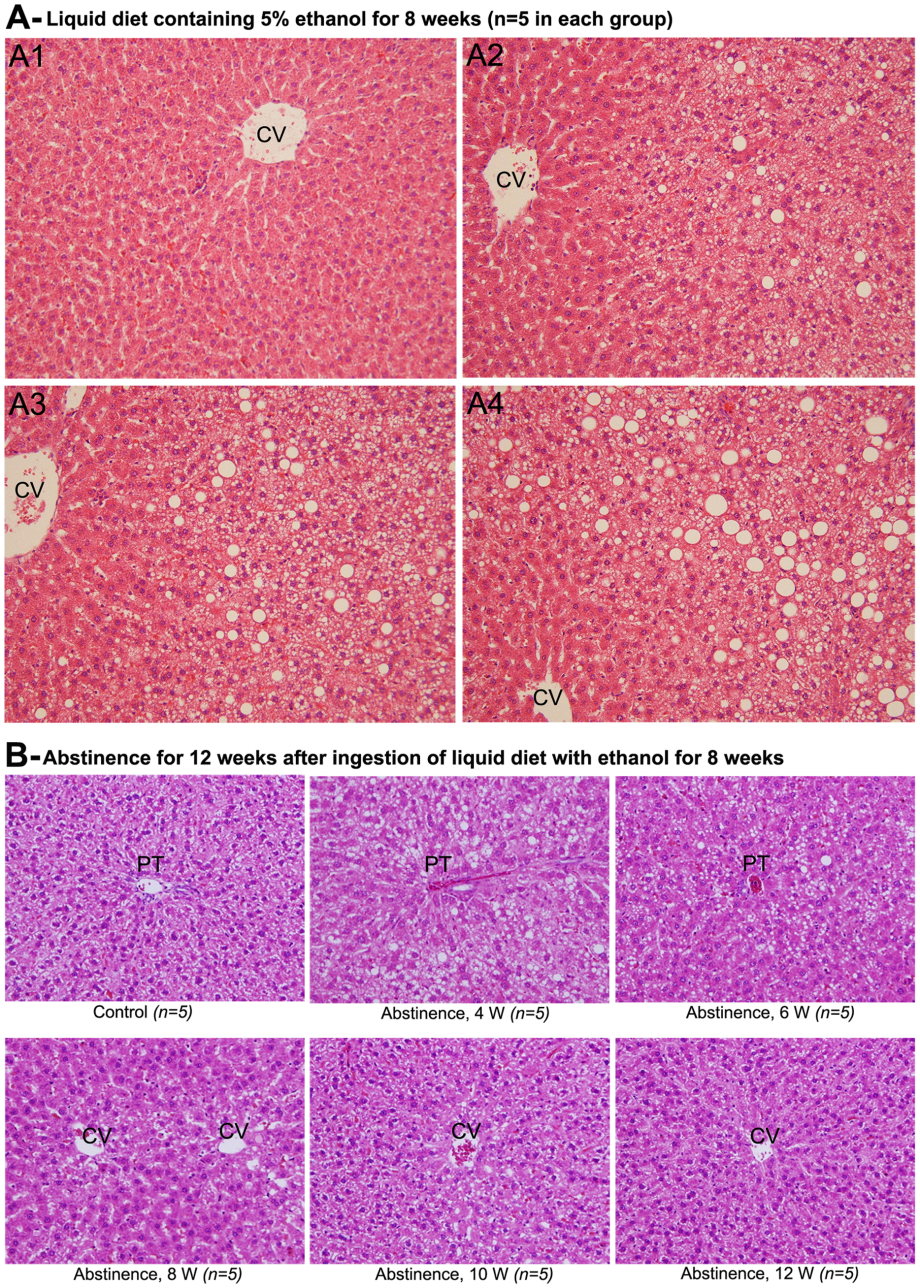
Histopathological alterations in rat liver during chronic administration of ethanol (H&E ×100). (A1) Histopathological changes were not observed in rats received control liquid diet. (A2), (A3) & (A4) Animals received liquid diet containing ethanol for 4, 6, and 8 weeks, respectively. Fatty degeneration was observed in all ethanol treated animals in increasing order from 4−8 weeks. (B) Abstinence of alcohol for 12 weeks (H&E x100). There was no alteration in the liver of rats received control liquid diet for 20 weeks. Fatty degeneration was ameliorated from 4−12 weeks with complete disappearance of steatosis at 12 weeks. CV−central vein, PT−portal triad.

The pathogenesis of alcoholic liver disease accompanied with hepatic fatty degeneration during chronic ingestion of ethanol is demonstrated through Fig. 3A1-4. The animals received control diet in which ethanol was isocalorically replaced with carbohydrate did not show any pathological alteration in the liver tissue after 8 weeks (Fig. 3-A1). The animals received ethanol-containing liquid diet showed moderate fat deposition in the pericentral areas without necrosis at 4th week (Fig. 3-A2). There was prominent fatty degeneration at 6th week with intermittent ballooning of hepatocytes (Fig. 3-A3). At 8th week of ethanol administration, the liver tissue showed enormous fatty degeneration and ballooning of hepatocytes in pericentral areas (Fig. 3-A4). However, hepatic fibrosis was completely absent and chronic administrations of ethanol in rats do not produce hepatic fibrosis.

The course of amelioration of ethanol induced alcoholic liver disease and fatty degeneration during a 12 week period of abstinence after chronic ingestion of ethanol through liquid diet for 8 weeks is demonstrated in Fig. 3B. The control animals received isocalorically replaced liquid diet did not show any pathological alteration in their livers. After 4 weeks of abstinence, the animals received liquid diet containing ethanol for 8 weeks showed marked fatty degeneration and ballooning of hepatocytes in pericentral areas. At 6 weeks of abstinence from alcohol, the livers showed numerous fat globules. However, hepatocyte ballooning was ceased or disappeared. At 8th weeks of abstinence, intermittent fatty degeneration was present. The livers showed almost normal architecture without much pathological alterations at 10th week of abstinence. At 12 weeks of abstinence, the animal livers were returned to normal architecture without any histopathological alterations.

### 5. Hepatic AA-AGE Reflects the Degree of Alcoholic Liver Disease during Chronic Administration of Ethanol and also in Abstinence

**Figure 4:**
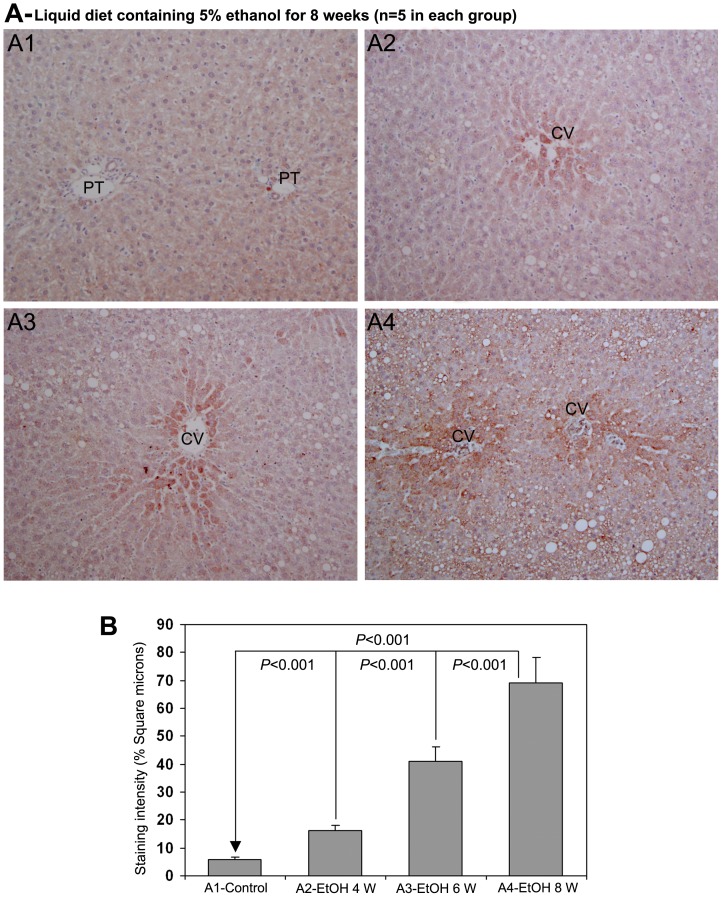
Immunohistochemical staining for AA-AGE in rat liver during chronic administration of ethanol (x100). (A1) Staining for AA-AGE was completely absent in rats received control liquid diet. (A2), (A3) & (A4) Animals received liquid diet containing 5% ethanol for 4, 6 and 8 weeks, respectively. Marked staining for AA-AGE in perivenular areas in increasing order from 4−8 weeks. CV−central vein, PT−portal triad. (B) Quantitative representation of the staining intensity of AA-AGE in A1–A4 stained sections (Mean ± S.D., *n = 5*).

The immunohistochemical staining for AA-AGE during chronic ingestion of ethanol through liquid diet is demonstrated in Fig. 4A. The staining for AA-AGE was completely absent in control rat livers (Fig. 4-A1). There was moderate staining of AA-AGE in the pericentral area of rat livers treated with ethanol for 4 weeks (Fig. 4-A2). On the 6th week of ethanol administration, the rat livers delineated strong staining of AA-AGE in the pericentral area (Fig. 4-A3). At 8 weeks of ethanol administration, the livers demonstrated intense staining of AA-AGE in the pericentral area and also in areas with fatty degeneration (Fig. 4-A4). The results of the quantitative evaluation of the staining intensity of AA-AGE are presented as percentage square microns in Fig. 4B. There was a significant increase (*P*<0.001) in the staining intensity of AA-AGE at 4, 6 and 8th weeks of ethanol administration when compared to non-treated controls. Furthermore, the staining intensity was also significantly higher (*P*<0.001) at 6th week compared to 4th week and at 8th week compared to 6th week (Fig. 4B).

**Figure 5:**
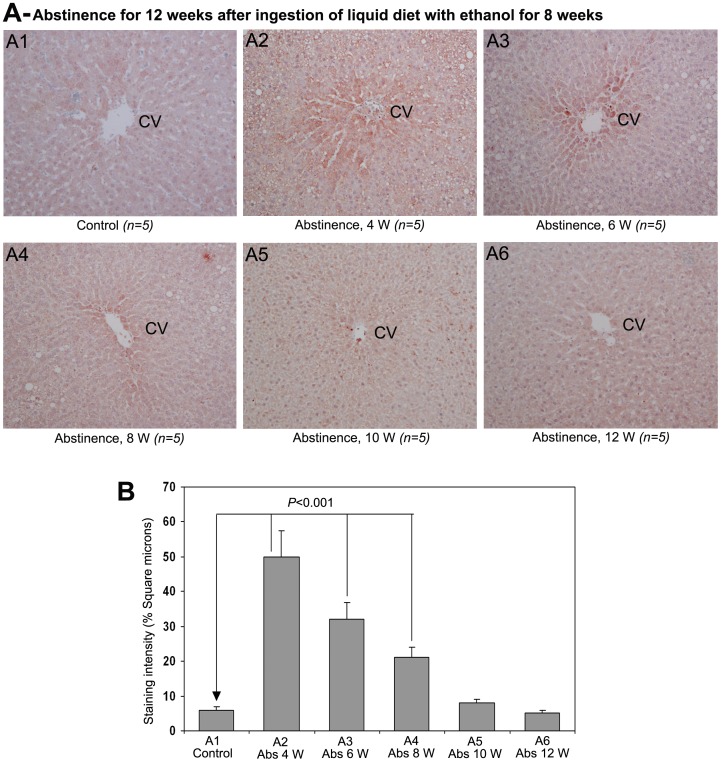
Effect of alcohol abstinence on AA-AGE (×100). (A) Staining for AA-AGE during abstinence of alcohol, 4−12 weeks. AA-AGE staining was absent in the liver of rats received control liquid diet for 20 weeks. Marked staining for AA-AGE was present in pericentral areas from 4−10 weeks of abstinence in sequentially decreasing order. Staining for AA-AGE was completely absent at 12 weeks of abstinence. CV−central vein (B) Quantitative representation of the staining intensity of AA-AGE in A1−A6 stained sections. The data are mean ± S.D. of five liver samples.

The staining of AA-AGE during a 12 week period of abstinence after 8 weeks of chronic administration of alcohol is demonstrated in Fig. 5A. The control animals received isocalorically replaced liquid diet did not show any staining of AA-AGE (Fig. 5A1). There was marked and intense staining of AA-AGE in pericentral area, hepatic chords and also in areas of fatty degeneration at 4 weeks of abstinence (Fig. 5A2). At 6th week of abstinence, the rat livers exhibited strong staining of AA-AGE, followed by moderate staining at 8th week. At 10th week of alcohol abstinence, there was only a mild staining of AA-AGE (Fig. 5A5). The staining of AA-AGE was completely absent at 12th week of abstinence (Fig. 5A6). The quantified data of the staining intensity of AA-AGE after alcohol abstinence is presented in Fig. 5B. A significant increase (*P*<0.001) was present in the staining intensity of AA-AGE at 4, 6 and 8th weeks after withdrawal of alcohol, but not on 10th and 12th weeks.

### 6. Staining of 4-HNE Correlates with AA-AGE and Degree of Alcoholic Liver Disease

**Figure 6:**
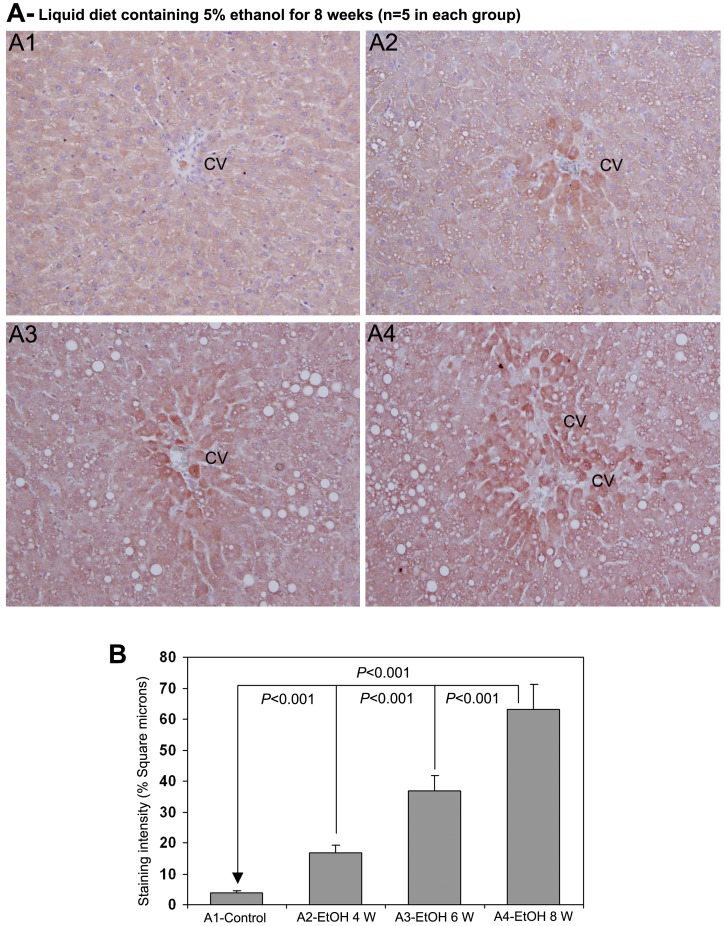
Immunohistochemical staining for 4-HNE in rat liver during chronic administration of ethanol (x100). (A1) Staining for 4-HNE was completely absent in rats received control liquid diet. (A2), (A3) & (A4) Animals received liquid diet containing 5% ethanol for 4, 6 and 8 weeks, respectively. Marked staining for 4-HNE in perivenular areas in increasing order from 4−8 weeks. CV−central vein. (B) Quantitative representation of the staining intensity of 4-HNE in A1−A4 stained sections (Mean ± S.D., *n = 5*).

The staining for 4-HNE during chronic administration of ethanol is demonstrated in Fig. 6A. The staining for 4-HNE was completely absent in control rat livers (Fig. 6-A1). There was significant staining of 4-HNE in the pericentral area of rat livers treated with ethanol for 4 weeks (Fig. 6-A2). At week 6th of chronic ethanol administration, the rat livers demonstrated marked staining of 4-HNE in the pericentral area and in hepatic chords (Fig. 6-A3). After 8 weeks of ethanol ingestion, the rat liver sections showed intense staining of 4-HNE in the pericentral area and also in areas with fatty degeneration (Fig. 6-A4). The staining pattern of AA-AGE and 4-HNE showed a distinct co-localization during the course of chronic ethanol administration leading the pathogenesis of alcoholic liver disease involving marked fatty degeneration. The quantitative evaluation of the staining intensity of 4-HNE after chronic administration of ethanol is presented in Fig. 6B. There was a significant increase (*P*<0.001) in the staining intensity of 4-HNE at 4, 6 and 8th weeks of ethanol administration compared with untreated controls. The staining intensity of 4-HNE was also significantly higher (*P*<0.001) at 6th week compared to 4th week and at 8th week compared to 6th week (Fig. 6B).

**Figure 7:**
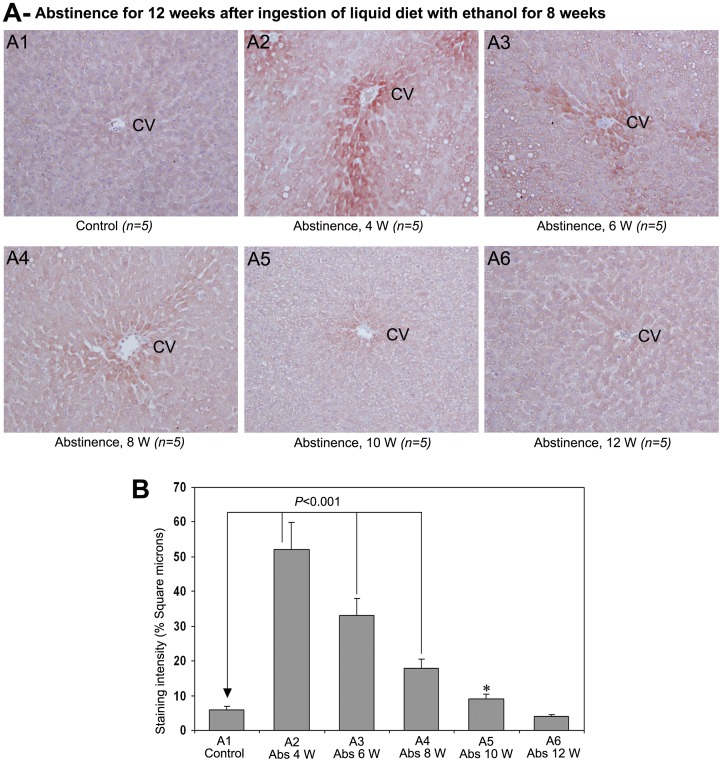
Effect of alcohol abstinence on 4-HNE (×100). (A) Staining for 4-HNE during abstinence of alcohol, 4−12 weeks. Staining for 4-HNE was absent in rats received control liquid diet for 20 weeks. There was marked staining for 4-HNE in pericentral areas from 4−10 weeks of abstinence in sequentially decreasing order. Staining for 4-HNE was completely absent at 12 weeks of abstinence. CV−central vein. (B) Quantitative representation of the staining intensity of 4-HNE in A1−A6 stained sections. The data are mean ± S.D. of five liver samples (*P<0.05).

The staining pattern of 4-HNE in rat liver sections during a 12 week period of abstinence after 8 weeks of chronic administration of ethanol is demonstrated in Fig. 7A. Staining for 4-HNE was completely absent in control animals received isocalorically replaced liquid diet (Fig. 7A1). There was intense and marked staining of 4-HNE in pericentral area extending to the centrilobular region and hepatic chords involving in areas of fatty degeneration at 4 weeks of abstinence (Fig. 7A2). At 6th week of abstinence, the rat livers demonstrated conspicuous staining of 4-HNE (Fig. 7A3). Even though there was strong staining of 4HNE at 8th week of abstinence, the staining pattern of 4-HNE was significantly reduced at 8th week compared to 6th week (Fig. 7A4). Mild staining of 4-HNE was present in the pericentral area at 10th week of abstinence (Fig. 7A5). The staining for 4-HNE was completely absent at 12 weeks of abstinence (Fig. 7A6). Figure 7B represents the quantitative evaluation of the staining intensity of 4-HNE after abstinence of alcohol. The staining intensity of 4-HNE was significantly higher (*P*<0.001) at 4, 6 and 8th weeks after withdrawal of alcohol when compared to non-treated control. The staining intensity was also higher (*P*<0.05) at 10th week but not at 12th week.

### 7. Marked Staining of 4-HNE Analogues with AA-AGE and ALD in Human

**Figure 8:**
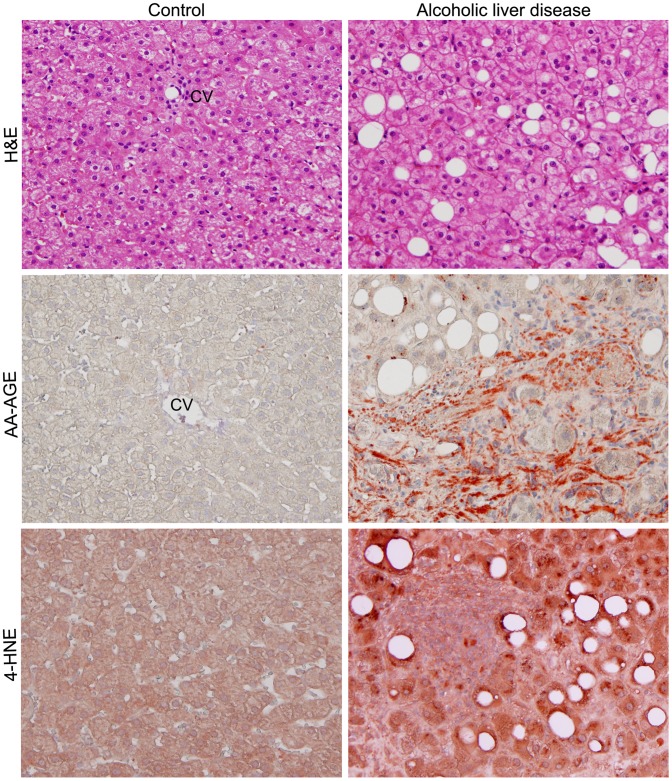
Histopathological evaluation for steatosis and immunohistochemical staining for AA-AGE and 4-HNE in liver biopsies from ALD patients (×200). H&E staining showed marked fatty degeneration. Strong staining for AA-AGE and 4-HNE was present in areas with severe fatty degeneration. Staining for AA-AGE and 4-HNE was also evident in areas without steatosis. CV−central vein.

Representative staining images for steatosis, AA-AGE, and 4-HNE of ALD patients along with control subjects are shown in Fig. 8. AA-AGE and 4-HNE staining was completely absent in control livers. Histopathological evaluation demonstrated severe fatty degeneration in all selected ALD patients. There was marked staining for AA-AGE and 4-HNE in the entire tissue specimen and the staining pattern and intensity were highly correlated (Fig. 8). This indicates an underlying mechanism of AA-AGE formation and ROS production or vice versa.

### 8. Treatment with AA-AGE Induced Oxidative Stress in Rat Hepatic Stellate Cells

**Figure 9:**
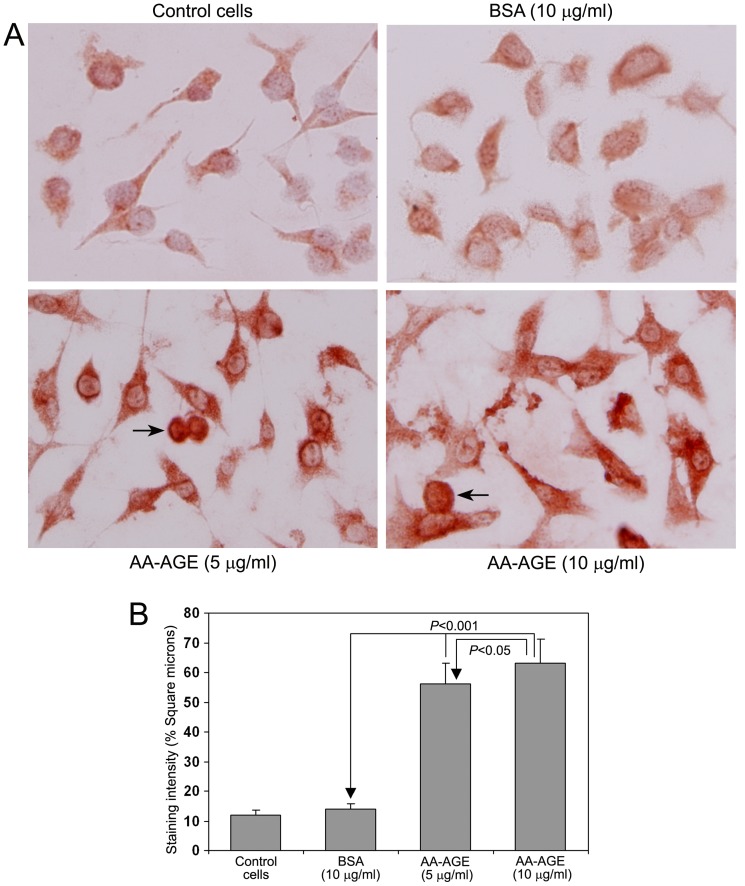
Immunohistochemical staining for 4-HNE in cultured rat hepatic stellate cells (×200). (A) Mild staining for 4-HNE was present in untreated control cells and cells treated with BSA. Marked staining for 4-HNE was present in stellate cells treated with both 5 µg/ml and 10 µg/ml AA-AGE. A few apoptotic cells (arrow) observed in cultures treated with both 5 µg/ml and 10 µg/ml AA-AGE. (B) Quantitative representation of the staining intensity of 4-HNE in cultured stellate cells (Mean ± S.D., *n = 5*).

Figure 9A demonstrates the role of AA-AGE in induction of oxidative stress and production of ROS in cultured rat hepatic stellate cells. The untreated control cells and cells treated with 10 µg/ml unmodified BSA showed mild staining for 4-HNE. Treatment with either 5 µg/ml or 10 µg/ml AA-AGE produced marked increase in the staining intensity of 4-HNE (Fig. 9A). Above 90% of cultured stellate cells treated with both 5 µg/ml and 10 µg/ml AA-AGE were viable. A few apoptotic cells were present in cultures treated with both 5 µg/ml and 10 µg/ml AA-AGE (marked with arrow). The quantitative data of the staining intensity of 4-HNE after treatment AA-AGE is presented in Fig. 9B. A significant increase (*P*<0.001) was observed in the staining intensity of 4-HNE in the cells treated with both 5 µg/ml and 10 µg/ml AA-AGE. The staining intensity of 4-HNE was significantly higher (*P*<0.05) in cells treated with 10 µg/ml AA-AGE compared to 5 µg/ml.

## Discussion

The present study was aimed to evaluate the role of AA-AGE during pathogenesis of alcoholic liver disease. We have previously observed that AA-AGE induces neurotoxicity and neuronal cell death [20]. Recently we have reported that toxic advanced glycation end-products (TAGE) play a significant role in the pathogenesis of diabetic vascular complications [26]. The aim of our present investigation was to show AA-AGE is more toxic to hepatocytes than NEL and also to demonstrate the association of AA-AGE and oxidative stress in the pathogenesis of alcohol induced fatty degeneration.

**Figure 10:**
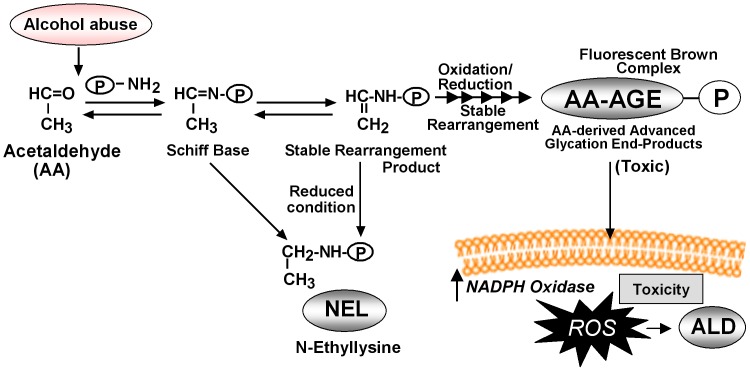
Schematic representation of the formation of AA-AGE from acetaldehyde, production of ROS, and pathogenesis of ALD. This scheme was prepared based on our current data and previous work.

In the current study, we evaluated the hepato-toxicity of AA-AGE and NEL on cultured rat primary hepatocytes. We observed a significant decrease in the viability of hepatocytes cultured with AA-AGE compared to the hepatocytes cultured with NEL. Furthermore, incubation of cortical neurons with AA-AGE produced a dose-dependent increase in neuronal cell-death, but not incubation with NEL [20]. The neurotoxicity of AA-AGE was neutralized by the addition of anti-AA-AGE antibody, but not by anti-NEL antibody, suggesting that AA-AGE is toxic to neuronal cells [20]. These data suggest that production of AA-AGE during alcoholism is not only toxic to hepatocytes but also to neuronal cells and probably many other cells inducing cellular and organ impairment. The NEL pathway for reaction of Amadori compounds may be physiologically relevant mechanism for averting production of AA-AGE and thus prevent the potential cellular toxicity arising from AA-AGE formation during alcoholism. However, during extensive oxidative stress in alcoholism, the stable rearranged products of the Schiff base could undergo further oxidation and reduction leading to the formation of AA-AGE (Fig. 10).

In order to examine the association of AA-AGE and pathogenesis of ALD, we induced ALD in rats using liquid diet containing ethanol for 4–8 weeks. Chronic administration of ethanol resulted in hepatic fatty degeneration in an increasing manner from 4–8 weeks. A characteristic feature of alcoholic liver injury is the predominance of lesions in the perivenular area, or zone 3, of the hepatic acinus [27]. One of the factors that may influence such selective perivenular cytotoxicity is zonal distribution of ethanol-metabolizing enzymes, mainly alcohol dehydrogenase (ADH) and CYP2E1. Although intralobular distribution of ADH is still controversial [28], [29], it is generally assumed that ADH is not inducible by ethanol [30], [31]. However it is well established that ethanol induces CYP2E1 prominently in the perivenular zone in rats and in man during chronic ethanol ingestion [31], [32]. This indicates that the amount of acetaldehyde metabolized from ethanol in perivenular hepatocytes is significantly higher than that in periportal hepatocytes during ethanol consumption.

This is the first study to demonstrate the intralobular distribution of AA-AGE during chronic ingestion of ethanol. In the present study we observed a marked increase in the staining for AA-AGE in pericentral areas of rat liver treated with ethanol. The staining intensity was significantly increased from 4 to 8 weeks indicating that the production of AA-AGE during chronic ethanol consumption is additive and also in parallel with the intensity of hepatic fatty degeneration and ALD. These data suggest that AA-AGE may play an important role to produce selective pericentral cytotoxicity in alcoholic liver disease.

In the present study, we have observed intense staining of AA-AGE and 4-HNE surrounding central vein during the pathogenesis of ALD as well as during early abstinence period. However, the fatty changes were mainly concentrated in the pericentral area. In both human and experimental animals, fatty changes usually occur in the pericentral area and not surrounding central vein. This could be due to the difference in the metabolic pattern within the hepatic lobule. Besides, the hepatocytes with fatty changes are in a dying stage and may not stain intensely like the healthy hepatocytes. Furthermore, the pathogenesis of ALD is not exclusively due to the formation of AA-AGE and 4-HNE, but only two factors contributing to the process.

In order to examine whether the AA-AGE accumulated during chronic ingestion of ethanol is eliminated after abstinence, the rats were administered with control liquid diet for a period 12 weeks after chronic administration of ethanol for 8 weeks. There was marked staining for AA-AGE in hepatocytes of perivenular region until week 8 during abstinence but significantly reduced by 10 weeks and completely disappeared at 12 weeks. Simultaneously, steatosis was also completely disappeared at 12 weeks with restoration of lobular architecture of hepatic tissue. These results suggest that the AA-AGE formed during chronic consumption of ethanol could be eliminated after abstinence which would help the impaired hepatic tissue to return to normal.

Acute and chronic consumption of ethanol increases the production of ROS through metabolism of alcohol, reduces cellular antioxidant levels, and enhances oxidative stress leading impairment of liver functions [33]. In the present study we have observed a significant decease of glutathione levels in the liver during chronic consumption of alcohol. The depleted glutathione levels were returned to normal at 10 weeks after the start of alcohol abstinence. Ethanol-induced oxidative stress plays a significant role in the pathogenesis of ALD and cirrhosis, which could induce hepatocarcinogenesis. Alcohol induces CYP2E1, which contributes ROS production through microsomal ethanol oxidizing system (MEOS) [34]. Furthermore, alcohol reduces the levels of potent antioxidants, that scavenge ROS and protects liver from ROS induced injury [35]. In the present study we have observed a dramatic increase in the staining of 4-HNE, which is a marker for oxidative stress and ROS, from 4–8 weeks of ethanol administration (Fig. 6). There was conspicuous correlation in the staining pattern of AA-AGE and 4-HNE during chronic administration of ethanol and also during the course alcohol abstinence. We also observed a striking correlation in the staining pattern of AA-AGE and 4-HNE in human ALD (Fig. 8). Furthermore, *in vitro* cell culture studies using rat hepatic stellate cells demonstrated that AA-AGE induces oxidative stress and produces ROS in culture (Fig. 9). These data indicate that AA-AGE induces the production of ROS and contributes to pathogenesis of ALD. Our previous studies [20] further support this theory.

A scheme for the formation of AA-AGE from acetaldehyde contributing to the pathogenesis of ALD is represented in Fig. 10. Alcohol is metabolized into acetaldehyde through alcohol dehydrogenase. Excessive production of acetaldehyde leads to the formation of Schiff base through amino group binding to a protein. The primary adduct thus formed when proteins are exposed to acetaldehyde under reducing conditions is N-ethyllysine (NEL). In our present study we demonstrated that NEL is not toxic to primary hepatocyte cultures (Fig. 1A). The Schiff base that bound to a protein not under reduced conditions forms a stable rearrangement product with a secondary amino group (Fig. 10). Such a protein bound product undergoes several oxidations and reductions and finally forms a fluorescent brown complex, the AA-AGE (Fig. 10). The AA-AGE thus formed binds to the cellular membrane through receptors, induces oxidative stress and produces ROS that triggers steatosis, hepatocyte ballooning and pathogenesis of ALD (Fig. 10). However, the exact molecular mechanisms of AA-AGE formation and generation of ROS are not clear. During chronic alcoholism ROS could also be generated through many other different mechanisms that promote the pathogenesis of ALD.

In conclusion, we demonstrated that AA-AGE is toxic to hepatocytes, but not NEL. Chronic alcohol consumption produces AA-AGE in pericentral hepatocytes from the metabolic products of ethanol, mainly acetaldehyde and induces oxidative stress leading to the production of ROS. Withdrawal of alcohol results in complete disappearance of fatty degeneration along with staining of both AA-AGE and 4-HNE suggesting that AA-AGE plays a role in pathogenesis of ALD.
